# Supplementary material for: Quantitative analysis questions the role of MeCP2 as a global regulator of alternative splicing
Source: PLoS Genet. 2020 Oct 13;16(10):e1009087. doi: 10.1371/journal.pgen.1009087 (PMC7584252; doi:10.1371/journal.pgen.1009087)
Supplement: S1 Table — (PDF) [file pgen.1009087.s001.pdf]

x	y	feature(y)	n	Pearson(r)	p-val	
$\Delta\Psi$ (*KO*-*WT*)	mCG (*WT*)	AE	101	0.071	0.371	GSE125660 [9]
		Exons		0.039	0.622	
		Introns		0.035	0.663	
$\Delta\Psi$ (*OE11x*-*WT*)		AE	73	-0.006	0.928	
		Exons		-0.085	0.227	
		Introns		0.114	0.102	
$\Delta\Psi$ (*OE4x*-*WT*)		AE	120	-0.040	0.636	
		Exons		-0.007	0.935	
		Introns		0.077	0.361	
$\Delta\Psi$ (*KO*-*WT*)	ΔmCA (*KO*-*WT*)	AE	57	-0.004	0.982	GSE128186 [17]
		Exons		-0.029	0.878	
		Introns		-0.298	0.104	
	ΔmCG (*KO*-*WT*)	AE	57	0.162	0.384	
		Exons		-0.114	0.542	
		Introns		-0.114	0.542	
$\Delta\Psi$ (Pv.8wk-Pv.1wk)	ΔmCA (Pv.8wk-Pv.1wk)	AE	489	0.066	0.121	GSE103214 [20]
		Exons		0.073	0.086	
		Introns		**0.129**	**0.002**	
	ΔmCG (Pv.8wk-Pv.1wk)	AE	489	0.036	0.404	
		Exons		0.022	0.605	
		Introns		0.059	0.167	
$\Delta\Psi$ (Pv.8wk-Pv.3wk)	ΔmCA (Pv.8wk-Pv.3wk)	AE	60	-0.047	0.725	
		Exons		0.002	0.989	
		Introns		0.108	0.419	
	ΔmCG (Pv.8wk-Pv.3wk)	AE	60	-0.045	0.735	
		Exons		-0.255	0.054	
		Introns		0.147	0.271	
$\Delta\Psi$ (Vip.8wk-Vip.1wk)	ΔmCA (Vip.8wk-Vip.1wk)	AE	408	-0.052	0.259	
		Exons		-0.098	0.035	
		Introns		-0.025	0.583	
	ΔmCG (Vip.8wk-Vip.1wk)	AE	408	-0.037	0.430	
		Exons		-0.027	0.558	
		Introns		-0.026	0.577	
$\Delta\Psi$ (Vip.8wk-Vip.3wk)	ΔmCA (Vip.8wk-Vip.3wk)	AE	527	0.034	0.450	
		Exons		-0.010	0.821	
		Introns		0.019	0.680	
	ΔmCG (Vip.8wk-Vip.3wk)	AE	527	-0.012	0.784	
		Exons		0.025	0.585	
		Introns		-0.068	0.136	
$\Delta\Psi$ (*DNMT-TKO*-*WT*)	mCG (*WT*)	AE	71	0.043	0.642	GSE64910 [18] GSE67867 [19]
		Exons		-0.076	0.415	
		Introns		-0.200	0.030	
$\Delta\Psi$ (*DNMT-TKO*-*WT*)	mCG (*WT*)	AE	71	-0.081	0.340	
		Exons		-0.153	0.068	
		Introns		0.107	0.207	
$\Delta\Psi$ (Pro-Gra)	ΔMeCP2 ChIP (Pro-Gra)	AE	205	0.007	0.924	GSE48307 [23]
		Exons		0.014	0.843	
		Introns		-0.044	0.535	
	ΔmCG (Pro-Gra)	AE	205	0.012	0.862	
		Exons		0.048	0.498	
		Introns		0.009	0.895	
