# Supplementary figures and images for: Quantitative analysis questions the role of MeCP2 as a global regulator of alternative splicing
Source: PLoS Genet. 2020 Oct 13;16(10):e1009087. doi: 10.1371/journal.pgen.1009087 (PMC7584252; doi:10.1371/journal.pgen.1009087)

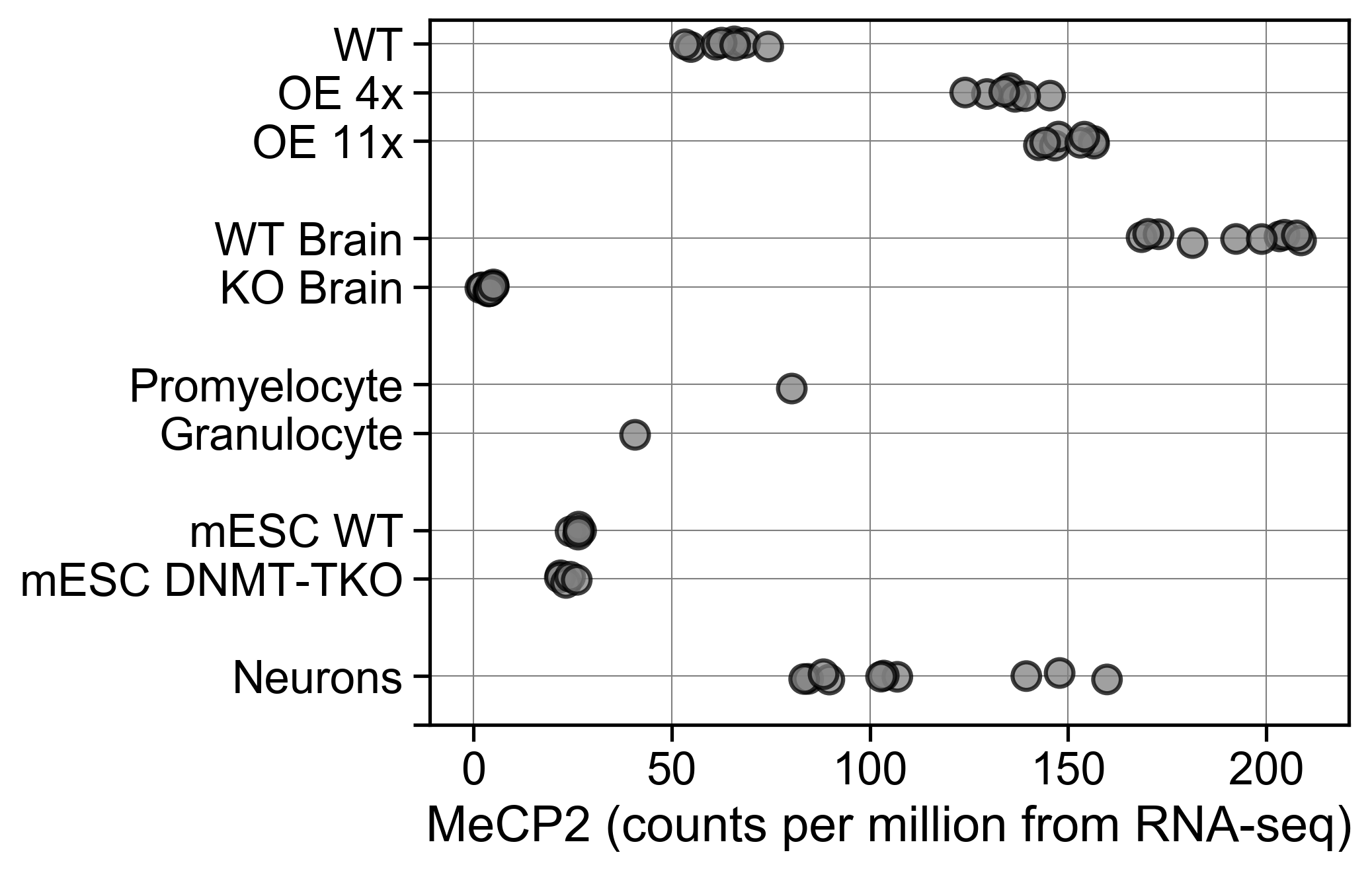

Supplement: S1 Fig — (TIF) [file pgen.1009087.s002.tif]

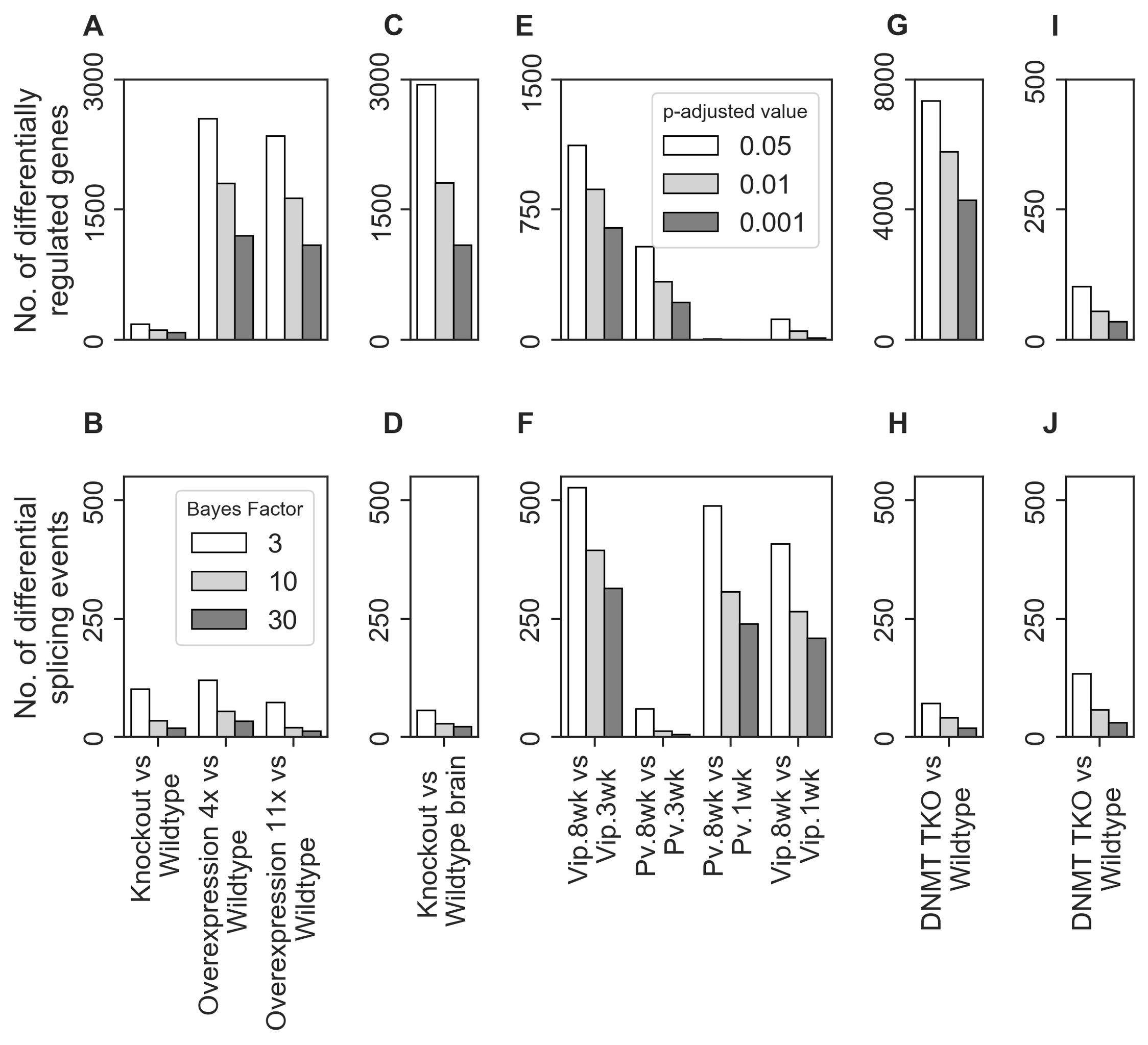

Supplement: S2 Fig — (A) Number of differentially regulated genes and (B) Number of differential splicing events in cultured neurons expressing multiple levels of MeCP2 [9] (C) Number of differentially regulated genes and (D) Number of differential splicing events in MeCP2 KO mouse brains compared to MeCP2 WT [17] (E) Number of differentially regulated genes and (F) Number of differential splicing events in developing mouse neurons [20] (G) Number of differentially regulated genes and (H) Number of differential splicing events in DNMT-TKO mESCs [19] (I) Number of differentially regulated genes and (J) Number of differential splicing events in DNMT-TKO mESCs [18]. (TIF) [file pgen.1009087.s003.tif]

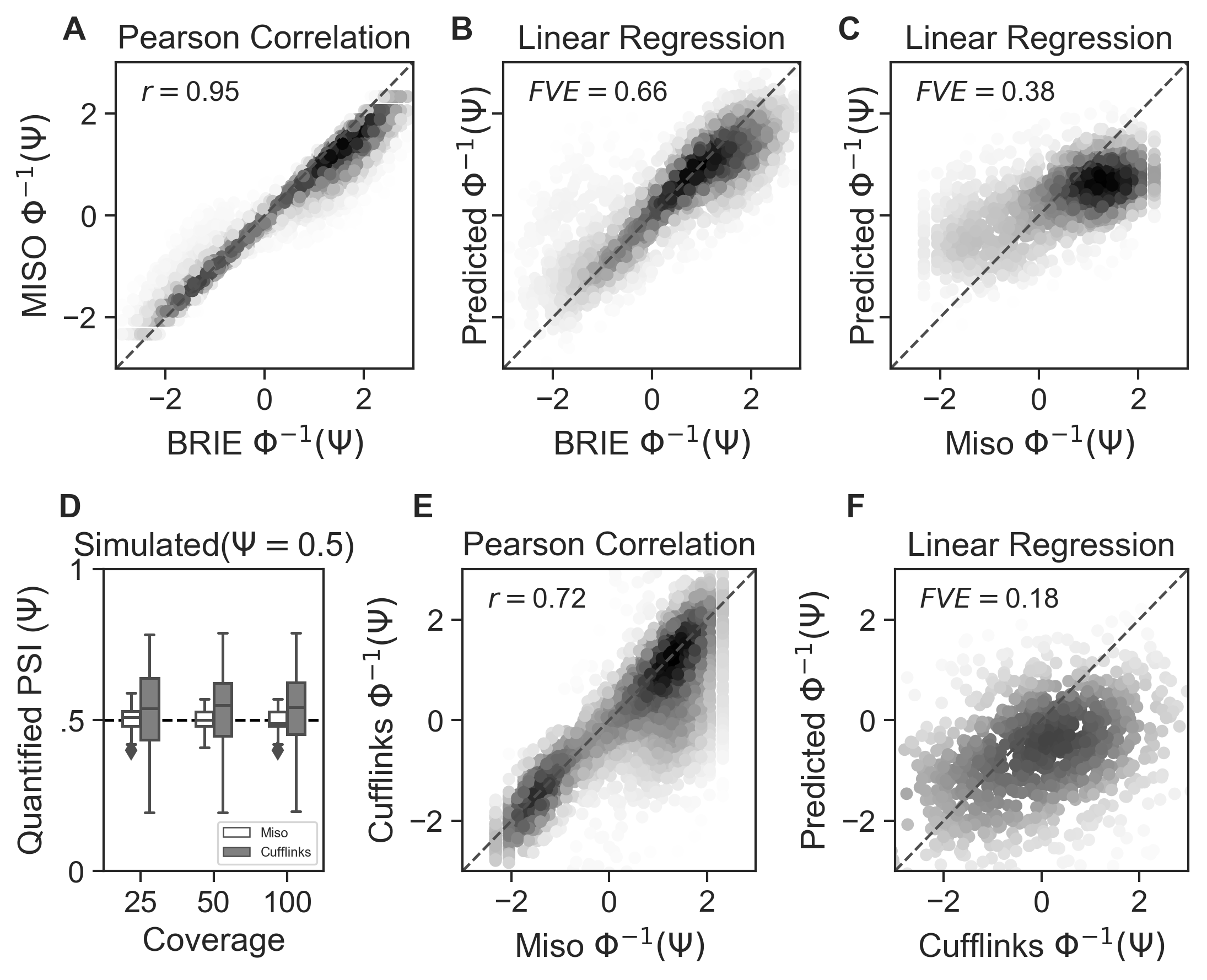

Supplement: S3 Fig — (A) Representative example of correlation between quantification methods BRIE and Miso. (B) Representative scatter plot showing prediction of splicing ratios using linear regression model learned from BRIE’s quantification and sequence features. (C) Representative scatter plot showing prediction of splicing ratios using linear regression model learned from Miso’s quantification and sequence features. (D) Comparison of splicing ratio estimation of known Ψ from simulated data at different coverage. (E) Representative example of correlation between quantification methods Cufflinks and Miso. (F) Representative scatter plot showing prediction of splicing ratios using linear regression model learned from Cufflinks’ quantification and sequence features. (TIF) [file pgen.1009087.s004.tif]

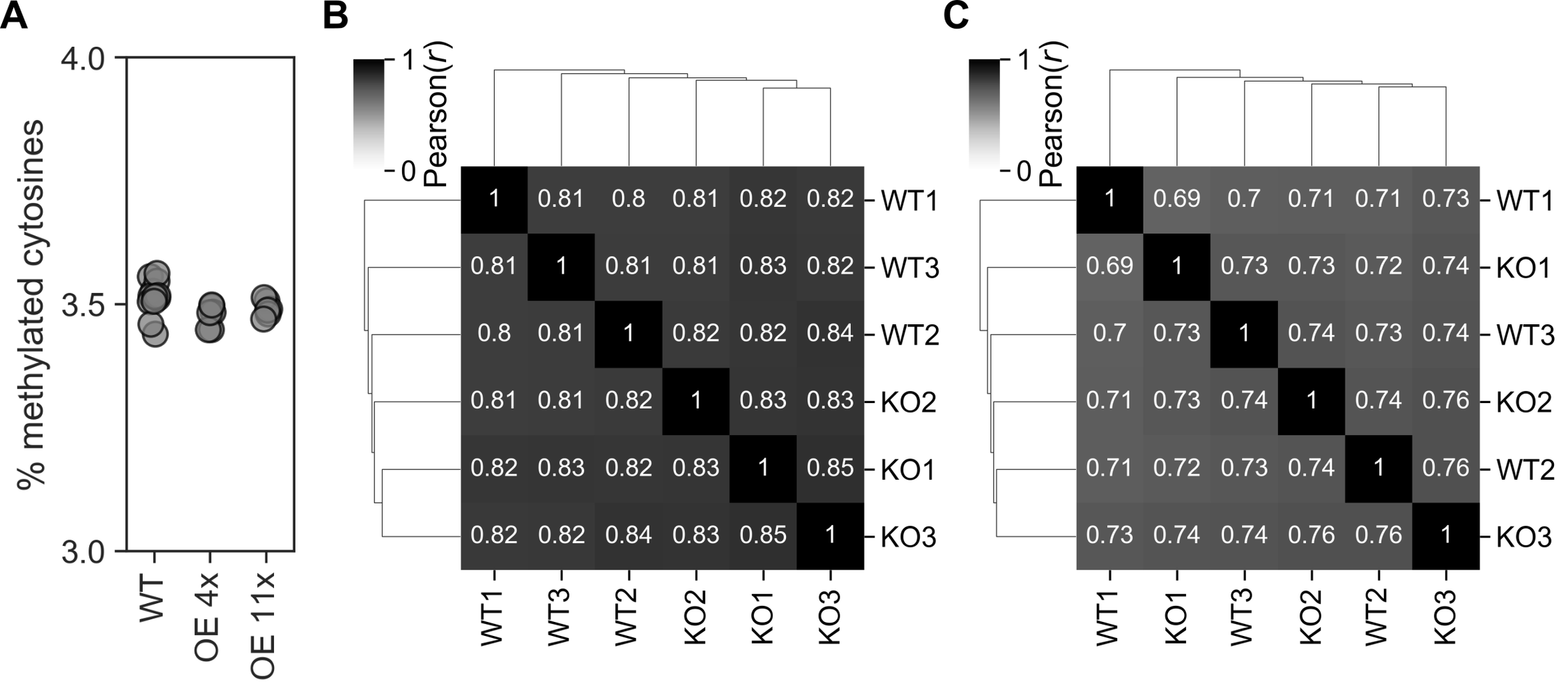

Supplement: S4 Fig — (A) Referencing results from our previous work [9]. High Performance Liquid Chromatography (HPLC) quantification of methylated cytosines in neurons expressing wild-type, 4 times and 11 times MeCP2. (B) CG and (C) CA methylation across introns quantified from bisulfite sequencing of WT and MeCP2 KO mouse brains (GSE128172 [17]). (TIF) [file pgen.1009087.s005.tif]

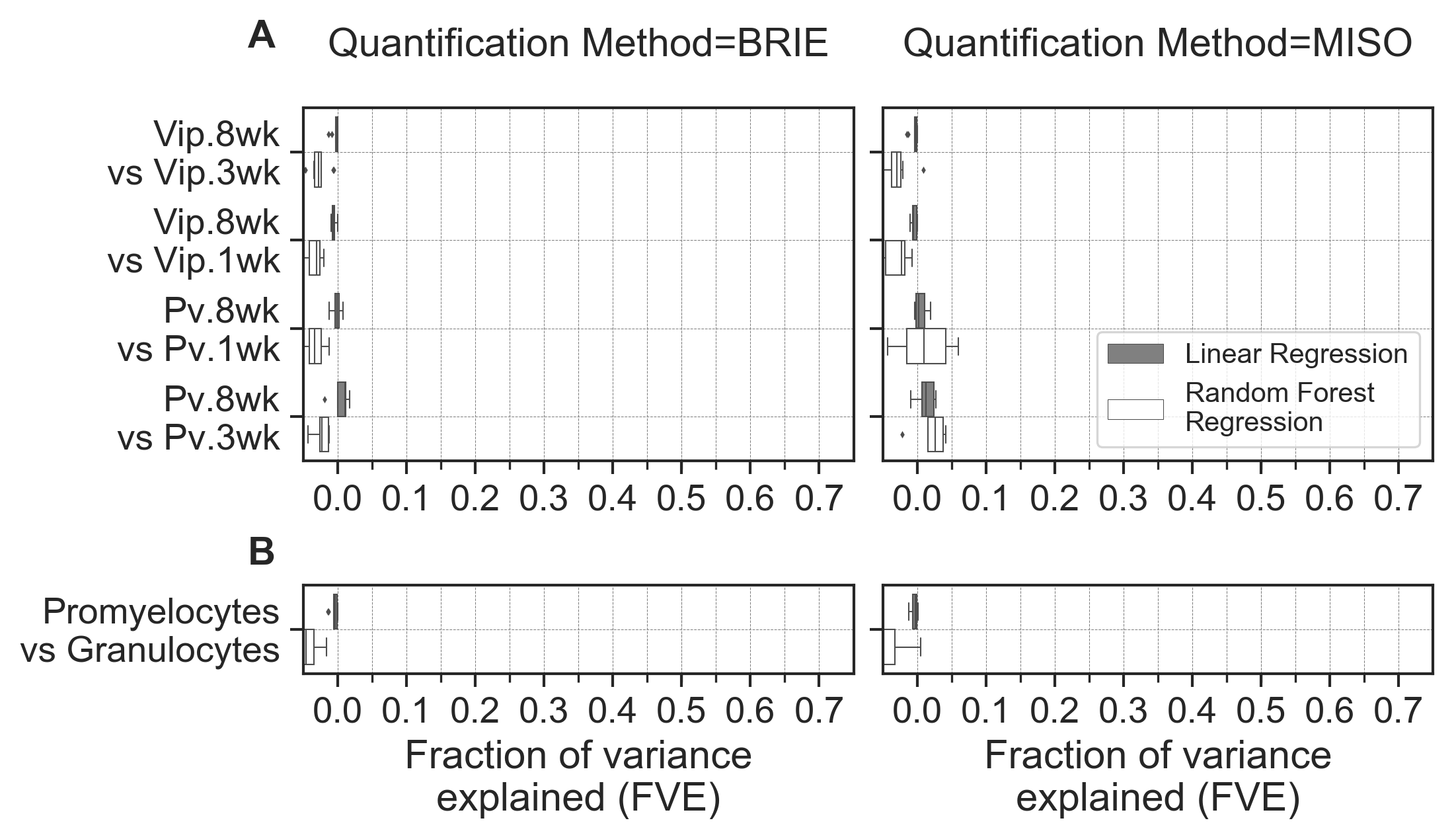

Supplement: S5 Fig — Fraction of variance explained by regressing DNA methylation features against differential splicing ratios in (A) developing mouse neurons [20](B) Promyelocytes and Granulocytes [14, 23]. (TIF) [file pgen.1009087.s006.tif]
