# Supplementary material for: Quantitative analysis questions the role of MeCP2 as a global regulator of alternative splicing
Source: PLoS Genet. 2020 Oct 13;16(10):e1009087. doi: 10.1371/journal.pgen.1009087 (PMC7584252; doi:10.1371/journal.pgen.1009087)
Supplement: Attachment — Submitted filename: Response_to_reviewers.pdf [file pgen.1009087.s008.pdf]

Detailed response to the reviews

Direct quotes from the referees' reports are given in *italics.*

Referee 1

We are grateful to this reviewer for his/ her appreciative reading of our work, which is judged to be *an important contribution towards understanding MeCP2 biology and its contribution to neuronal function.* The reviewer also posed some specific questions, addressed below:

- *the authors investigate the contribution of MeCP2 to splicing through analysis of transcriptomic data from cells with different levels of MECP2 but constant total methylation data. They find that MeCP2 primarily functions as a regulator of gene expression and has minimal effect on splicing. In these comparisons, the authors mention that global DNA methylation is constant, however there is no analysis provided that supports that conclusion.* We apologise for this omission, the data on the constancy of global DNA methylation is now shown in S4 Fig A
- *Differential methylation could be restricted to a small number of regions and the authors need to exclude that the alternative splicing events (even if low in number) are not occurring at these differentially methylated sites.* This is a valid point, and cannot be addressed in the current LUHMES data set. We have therefore expanded our analysis to another recent data set (Boxer et al, reference 17) which provides paired RNA-seq and WGBS-seq in WT and MeCP2 KO mouse brains at high sequencing depth. Analysis on this data set showed that: a) methylomes in WT and KO brains are very strongly correlated, in fact with correlation levels indistinguishable from the ones between biological replicates (S4 Fig B); b) DNA methylation changes at differentially spliced genes are in general minimal (S1 Table). Taken together, these observations strengthen our conclusions that MeCP2 is not a global regulator of splicing, although they do not rule out a possible gene- and tissue- specific role.
- *The authors utilise DNMT-TKO ES cells to address how much differential methylation would influence splicing, however there could be profound differences between ES cells and neurons in DNA-methylation-mediated splicing regulation, and this needs to be addressed.* The reviewer is right; we did not intend the DNMT-TKO analysis to be a replacement for the missing DNA methylation data in the LUHMES cells, a problem now addressed via the use of the Boxer et al data. Rather, the DNMT-TKO analysis is meant to probe global role of DNA-methylation in splicing regulation in a different system where MeCP2 is not expressed at constitutively high levels. We have further clarified this in lines 47-53.

- *...This also assumes that DNA methylation and binding of MeCP2 is constant across the entire genome and over all exon/intron boundaries - which is not the case. Therefore, a context-dependent role can not be excluded where MeCP2 activity or DNA methylation would influence splicing only if certain criteria are met. E.g. only a at few splicing sites where high MeCP2 binding or high DNA methylation levels are present to influence splicing. I am not sure if the model applied by the authors fully takes these variations in consideration.* Indeed, our analysis primarily aims at discounting a global regulatory role; we cannot rule out that MeCP2 or DNA methylation will influence splicing under specific circumstances at specific loci. This is discussed in the second paragraph of the discussion, and further emphasised by our changing the manuscript's title. It should however be pointed out that our analysis on the Boxer data shows that changes in splicing in MeCP2 KO brains are not associated with changes in methylation, thus removing the assumption of constant DNA methylation.

Referee 2

We thank the reviewer for the entirely positive reading of our manuscript. The reviewer has a single comment:

- *My main question is biological: I understand using the TKO cells as they are devoid of DNA methylation, but aren't they also quite low for MeCP2 expression? How can we rule out that there is no effect on splicing because MeCP2 is absent or low? If the authors could show the FPKMs for MeCP2 in ES cells, in comparison to the cells used in the Nat Comm paper (promyelocytes/granulocytes), that would help interpretation.* Indeed, ES cells express lower levels of MeCP2 compared to the other cell types considered (expression levels of MeCP2 are now shown in S1 Fig). The rationale for selecting the TKO cells was in fact to evaluate the role of DNA methylation independently of MeCP2, this is now further clarified in lines 47-52.

Referee 3

We thank the reviewer for the positive reading of our manuscript. The reviewer raises the following concerns:

- *many of the datasets used in the study are not "high quality". For example, some of the ESC data (both wt and KO) from the Ast lab are sequenced at a depth of 40M reads (SE, 50bp) which would be insufficient or barely sufficient for proper AS data analyses.*

Similarly, the brain neuron RNA-seq data is sequenced at 30M or less (50bp SE). Vip neuron data are sequenced at 20M, which is fairly low and insufficient for any meaningful AS analysis. The authors should provide sequencing depth for each sample (ideally in millions of mapped reads) in Table 1. We thank the reviewer for raising this important point. Concerning the TKO data, we use the Domcke et al RNA-seq for splicing analyses, which is sequenced at over 100M reads (the data from the Ast lab is used for WGBS-seq in the same cell type). It is true that the early developmental time points in the Stroud et al data sets have lower coverage due to technical limitations, and this will limit the power of AS analyses (although the Bayesian methods employed should not introduce false positives). Nevertheless, we felt that the developmental data set was important due to the gradual increase in mCA observed in development, and therefore it was included. We now caveat about the lower coverage in lines 68-69. We have also added depth information in Table 1 as requested.

- *It should also be explained better why the selected samples were chosen and how their suitability for AS analyses was determined. As sequencing depth plays a major role in AS detection, a few sentences on the role and effects of depth in the current analyses would be most welcome.* We thank the reviewer for raising this point and have reflected on it by providing additional information and adjusting the text in lines 42-75, specifically 67-73.
- *The y axes in figures S1B and S1D need to be adjusted. I don't think it is necessary to use the same scale for differential gene expression and differential splicing. I understand that the authors want to show that the number of differential splicing events is minimal when compared to the number of differentially expressed genes, however it would be much better to represent the splicing data on a separate scale (ie. 1-100 or something like that).* We thank the reviewer for the suggestion, which has been implemented in S2 Fig now.
- *It would be beneficial if the authors could produce a figure with MeCP2 expression levels across different tissues / samples used in the current manuscript.* S1 Fig now shows expression levels of MeCP2 across data sets
- *Finally, as this study only discusses the global impact of MeCP2 on AS and does not try to confirm or refute potential locus specific AS events impacted by MeCP2 binding, the title needs to be adjusted. Something like: "Quantitative analysis questions the role of MeCP2 as a genome-wide (global?) regulator of alternative splicing" would be a better fit.* We followed the suggestion and changed the manuscript's title.
